# Strongly interacting Rydberg atoms in synthetic dimensions with a magnetic flux

**DOI:** 10.1038/s41467-024-46823-6

**Published:** 2024-03-27

**Authors:** Tao Chen, Chenxi Huang, Ivan Velkovsky, Kaden R. A. Hazzard, Jacob P. Covey, Bryce Gadway

**Affiliations:** 1 Department of Physics, University of Illinois at Urbana-Champaign, Urbana, IL 61801-3080 USA; 2 Department of Physics and Astronomy, Rice University, Houston, TX 77005 USA; 3 Rice Center for Quantum Materials, Rice University, Houston, TX 77005 USA; 4 Department of Physics, University of California, Davis, CA 95616 USA

**Keywords:** Quantum simulation, Atomic and molecular physics

## Abstract

Synthetic dimensions, wherein dynamics occurs in a set of internal states, have found great success in recent years in exploring topological effects in cold atoms and photonics. However, the phenomena thus far explored have largely been restricted to the non-interacting or weakly interacting regimes. Here, we extend the synthetic dimensions playbook to strongly interacting systems of Rydberg atoms prepared in optical tweezer arrays. We use precise control over driving microwave fields to introduce a tunable *U*(1) flux in a four-site lattice of coupled Rydberg levels. We find highly coherent dynamics, in good agreement with theory. Single atoms show oscillatory dynamics controllable by the gauge field. Small arrays of interacting atoms exhibit behavior suggestive of the emergence of ergodic and arrested dynamics in the regimes of intermediate and strong interactions, respectively. These demonstrations pave the way for future explorations of strongly interacting dynamics and many-body phases in Rydberg synthetic lattices.

## Abstract (web summary)

Weak and non-interacting systems have been previously explored in synthetic dimensions. Here the authors demonstrate strong atomic interaction in synthetic dimensions using an array of Rydberg atoms.

## Introduction

Analog quantum simulation in atomic, molecular, and optical systems has seen tremendous growth over the past decades. Recently, a flurry of activity has expanded analog simulations through synthetic dimensions^1–3^, where dynamics occurs not in space but in alternative degrees of freedom such as spin. Since the first proposals a decade ago^4,5^, the synthetic dimensions approach has permeated photonic and atomic physics experiment, with demonstrations in systems of atomic hyperfine states^6,7^, metastable atomic “clock” states^8–10^, atomic momentum states^11–13^, trap states^14,15^, photonic frequency modes^16^, orbital angular momentum modes^17^, time-bin modes^18^, and more. The realization of synthetic dimensions in these diverse platforms has led to a plethora of new simulation capabilities^1,2^. However, studies have been almost entirely restricted to the non-interacting regime, with just a handful probing collective mean-field interactions in synthetic dimensions^19–24^ and only one recent report of strongly correlated dynamics in synthetic dimensions^25^.

Several years ago, arrays of trapped molecules and Rydberg atoms were proposed^26–28^ as an alternative paradigm for exploring synthetic dimensions with strong interactions. In this approach, one starts with a dipolar spin system in which interactions naturally play a significant role^29–31^. Then, by introducing tailored microwaves that drive transitions between internal states in a way that mimics the hopping structure of a lattice tight-binding model, the spin system is transformed into a playground for exploring the dynamics of strongly interacting matter in a synthetic dimension. In the past year, the team of Kanungo and co-workers have demonstrated the first Rydberg synthetic lattice^32^, engineering and probing topological band structures formed from the Rydberg levels of individual Sr atoms. While this demonstration^32^ has laid the foundation for future developments of Rydberg and molecular synthetic lattices (see also ref. ^33^ for steps towards molecular synthetic dimensions, as well as related early work in Rydbergs and molecules^34,35^), it lacked the key ingredient motivating the use of Rydberg atoms: strong dipole–dipole interactions.

In this paper, we extend the capabilities of Rydberg synthetic dimensions by engineering an internal-state lattice with a tunable artificial gauge field^36–41^ for small arrays of strongly interacting atoms^42^. We show that the promising results of ref. ^32^, wherein continuous microwave coupling is performed for single Rydberg atoms excited from a bulk sample, extend directly to the real-time dynamical control of atoms prepared in optical tweezer arrays^42,43^. The control of the artificial gauge field in the synthetic dimension follows naturally from our phase-coherent control of the driving microwave fields. Finally, strong nearest-neighbor interactions in the synthetic dimension lead to strong modifications of the population dynamics as well as the observation of atom-atom correlations. This work paves the way for future explorations of strongly correlated dynamics and phases of matter in Rydberg and molecular synthetic dimensions.

## Results

### Implementation of U(1)-flux Rydberg lattice

**Fig. 1:**
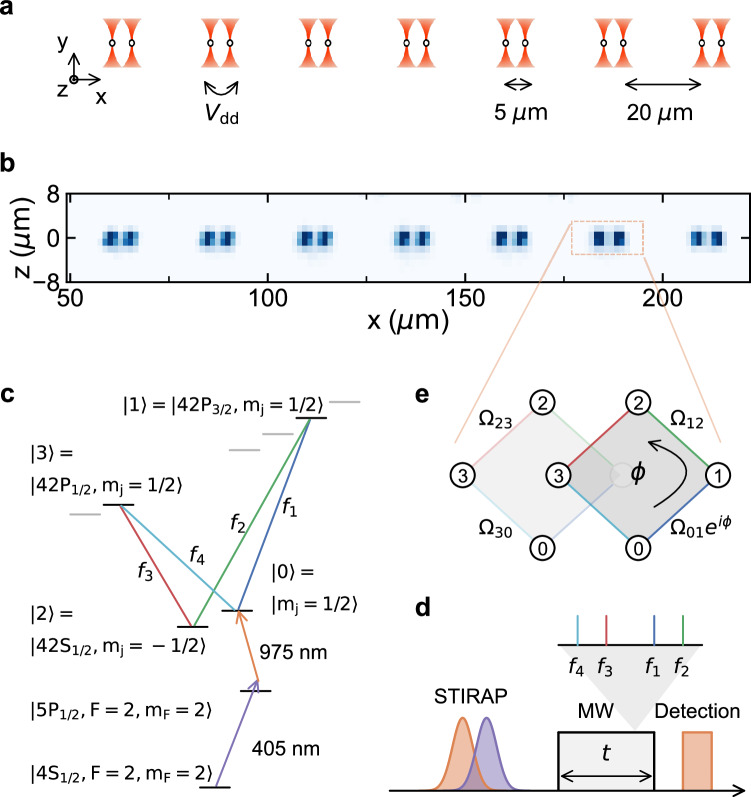
Rydberg synthetic dimensions for tweezer-trapped atom arrays. **a** A pairwise array of optical tweezer traps is used to initialize isolated pairs of ^39^K atoms. **b** Averaged (over 1000 shots) fluorescence image of the atom array. **c**, **d** Ground state atoms are transferred via STIRAP to the $\vert 42{S}_{1/2},{m}_{J}=1/2\rangle$ Rydberg level and then exposed to microwaves (with frequencies *f*_1−4_) to simultaneously drive multiple transitions between Rydberg levels. Dynamics of the Rydberg state populations is achieved by state-selective depumping and the imaging of ground state atoms. **e** The engineered synthetic Rydberg lattice, a diamond plaquette with flux *ϕ* that is tuned via the microwave phases.

Our experiments begin by probabilistically loading ^39^K atoms^44,45^ into optical tweezer arrays as depicted in Fig. 1a, b, nondestructively imaging the atoms for subsequent post-selection, and cooling the atoms by gray molasses^45,46^. We optically pump the atoms (with a quantization *B*-field of ≈27 G along the *z-*axis) to a single ground level $\vert 4{S}_{1/2},\, F=2,\, {m}_{F}=2\rangle$ with an efficiency of 98(1) %, and then we further cool the atoms by trap decompression to ≈4 *μ* K. We then suddenly turn off the confining tweezer trap.

The atoms undergo a fixed free release time of 5 *μ*s, during which all of the dynamics in the Rydberg synthetic lattice occurs. The atoms are promoted to an initial Rydberg level, undergo microwave-driven dynamics between Rydberg levels, and are de-excited in a manner that allows for Rydberg state-specific readout. Following de-excitation, ground-state atoms are recaptured in the trap and imaged with high fidelity. Atoms remaining in the Rydberg levels are weakly anti-trapped by the tweezers, and are effectively lost between the initial and final images. This bright/dark discrimination between ground and Rydberg levels, combined with state-selective de-excitation, allows us to study the state-resolved dynamics of the Rydberg level populations.

The initial excitation to the Rydberg level $\vert 0\rangle \equiv \vert 42{S}_{1/2},{m}_{J}=1/2\rangle$ is accomplished via two-photon ("lower leg,” ~405 nm, and “upper leg,” ~975 nm) stimulated Raman adiabatic passage (STIRAP) via the $\vert 5{P}_{1/2},F=2,{m}_{F}=1/2\rangle$ intermediate state^47,48^. The averaged one-way STIRAP efficiency is 94(1)%^49^. After populating this initial state, we turn on a set of microwave tones that allow atoms to “hop” between the sites of an effective lattice in the “synthetic dimension” spanned by the Rydberg levels^4,26,32^.

As shown in Fig. 1c–e, we identify the sites of the synthetic Rydberg lattice with the atomic Rydberg levels as $\left\vert 0\right\rangle \equiv \vert 42{S}_{1/2},{m}_{J}=1/2\rangle$, $\left\vert 1\right\rangle \equiv \vert 42{P}_{{{{{{{{\rm{3/2}}}}}}}}},{m}_{J}=1/2\rangle$, $\left\vert 2\right\rangle \equiv \vert 42{S}_{1/2},{m}_{J}=-1/2\rangle$, and $\left\vert 3\right\rangle \equiv \vert 42{P}_{1/2},{m}_{J}= 1/2\rangle$. A single flux plaquette is formed by adding microwave tones that resonantly drive four pairwise transitions within this set of states.

The effective single-atom Hamiltonian is given by

1 $$H=\mathop{\sum}\limits_{\langle i,j\rangle }\frac{{\Omega }_{ij}}{2}\,{\hat{c}}_{j}^{{{{\dagger}}} }{\hat{c}}_{i}+{{{{{{{\rm{h}}}}}}}}.{{{{{{{\rm{c}}}}}}}}.=\frac{\Omega }{2}\mathop{\sum}\limits_{\langle i,j\rangle }{e}^{i{\phi }_{ij}}\,{\hat{c}}_{j}^{{{{\dagger}}} }{\hat{c}}_{i}+{{{{{{{\rm{h}}}}}}}}.{{{{{{{\rm{c}}}}}}}}.\,,$$

where the nearest-neighbor tunneling terms are related to the amplitudes (*A*_*i*_) and phases (*φ*_*i*_, at the atoms) of the different microwave tones *f*_*i*_ as ${\Omega }_{01}\propto {A}_{1}{e}^{i{\varphi }_{1}}$, ${\Omega }_{12}={\Omega }_{21}^{*}\propto {A}_{2}{e}^{-i{\varphi }_{2}}$, ${\Omega }_{23}\propto {A}_{3}{e}^{i{\varphi }_{3}}$, and ${\Omega }_{30}={\Omega }_{03}^{*}\propto {A}_{4}{e}^{-i{\varphi }_{4}}$. The magnitudes of these nearest-neighbor hopping terms are calibrated based on pairwise Rabi dynamics^49^ and are set to a common value Ω. The relative phase of each tone at the atoms is controllable by the source phase, and in particular we set the overall plaquette flux *ϕ* via the source phase of the *f*_1_ tone (calibrated by fitting to the dynamics of singles after an evolution time of ~*h*/Ω^49^). This flux control by external fields complements recent real-space studies of interaction-derived Peierls phases^50^.

**Fig. 2:**
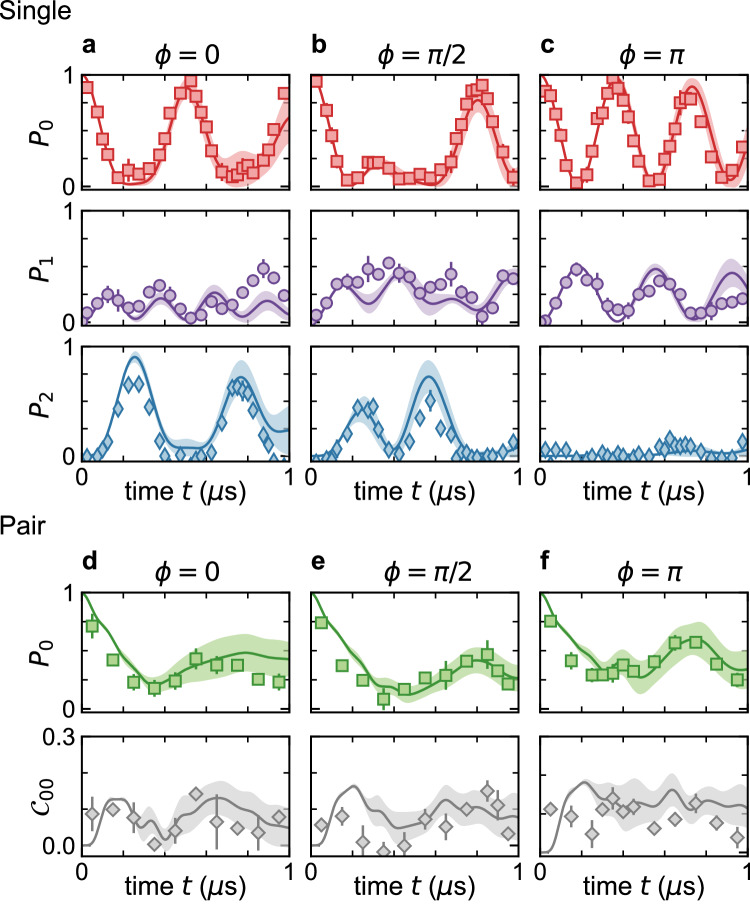
Dynamics of Rydberg atoms and pairs of atoms in a synthetic flux plaquette. **a**–**c** For flux *ϕ* of (**a**) 0, (**b**) *π*/2, and (**c**) *π*, we plot the average populations of single atoms at the synthetic sites $\left\vert 0\right\rangle$, $\left\vert 1\right\rangle$, and $\left\vert 2\right\rangle$ (top to bottom), corrected for state preparation and measurement infidelities^49^. **d**–**f** For the same flux values as above, but for the case of interacting atom pairs, we plot (top) the average atom population at site $\left\vert 0\right\rangle$, *P*_0_, and (bottom) the two-atom correlator *C*_00_. The error bars for all data are the standard error of multiple independent data sets taken under the same condition. The typical singles dataset is derived from roughly 200 post-selected images while pairs relate to roughly 50 post-selected images. The theory curves and confidence intervals for all the singles and pairs data are based on Eqs. (1) and (2) and the calibrated parameter values and uncertainties^49^ (including effects of SPAM and positional fluctuations), as detailed in the text. All figure data relate to parameter values of Ω/*h* = 1.92(6) MHz and *V*/*h* = 3.44(8) MHz for pairs.

Figure 2 displays the dynamics of the state populations (starting from the $\left\vert 0\right\rangle$ state at *t* = 0). The populations are corrected for state preparation and measurement (SPAM) errors related to the STIRAP infidelity and Rydberg-vs.-ground discrimination infidelity^49^. The $\left\vert 0\right\rangle$ state is measured by direct depumping by the “upper leg” (975 nm) STIRAP laser after some evolution time. To access the $\left\vert 1\right\rangle$ state, which shares identical population dynamics in this model as the $\left\vert 3\right\rangle$ state, we first apply a *π* pulse on the $\left\vert 0\right\rangle$ to $\left\vert 1\right\rangle$ transition prior to depumping. To access the $\left\vert 2\right\rangle$ state, which is quite close in energy to the $\left\vert 0\right\rangle$ state, we simply apply a strong (high-bandwidth) depumping pulse to measure the combined population of $\left\vert 0\right\rangle$ and $\left\vert 2\right\rangle$, *P*_0+2_. We then extract the $\left\vert 2\right\rangle$ state population as *P*_2_ = *P*_0+2_ − *P*_0_. For single atoms we generally find good agreement with the population dynamics for the examined flux values of Fig. 2 (a) 0, (b) *π*/2, and (c) *π* (uncertainties of 0.02*π*). The changing timescales for *P*_0_ recurrence reflects the flux-tuned spectral gaps of the plaquette energy spectrum. One stark signature seen in Fig. 2c, for *π* flux, is the absence of population appearing at state $\left\vert 2\right\rangle$, which results from destructive interference of the clockwise and counterclockwise pathways. To note, this interference effect relates to the phenomena of Aharonov–Bohm caging on a single plaquette^51,52^.

### Role of the dipolar interactions

The dynamics of lone atoms in Fig. 2a–c verifies our faithful implementation of the single-particle synthetic lattice and flux control. In Fig. 2d, e, we use isolated pairs of atoms to investigate how strong inter-particle interactions enrich the dynamics. The principal interactions between Rydberg atoms in this system involve long-ranged (1/*r*^3^, with *r* the inter-particle spacing) dipolar exchange^30^. In our system, having a uniform magnetic quantization field that breaks the degeneracy of different Rydberg *m*_*J*_ sublevels and is aligned at an angle *θ* = *π*/2 with respect to the displacement vectors between pairs of atoms, the primary interactions to consider are resonant dipolar-exchange terms of the form ${\vert i\rangle }_{m}{\vert \,\, j\rangle }_{n}\leftrightarrow {\vert \,\, j\rangle }_{m}{\vert i\rangle }_{n}$, or “flip–flop” interactions.

This interaction can be viewed as the anti-correlated hopping (in the synthetic lattice) of excitations on neighboring atoms. These particular Δ*ℓ* = 0 dipolar terms, which conserve the net populations of the individual states, also naturally conserve the total energy in a spatially uniform system and thus result in resonant exchange dynamics^29,53^. Based on our chosen Rydberg state assignments for the synthetic sites, these flip-flop interactions are only between nearest neighbors in the internal space. However, because the interactions depend on the Rydberg state details, they are not uniform along the synthetic dimension (i.e., they do not possess discrete translation symmetry). For pairs of atoms spaced at a distance of 5 *μ*m [Fig. 1a, b], the resonant dipolar exchange energies can be enumerated as {*V*_01_, *V*_12_, *V*_23_, *V*_30_} ≈ {2, − 0.5, 1, − 1}*V*, where *V*/*h* = 3.44(8) MHz^49^. Because we operate at a modest magnetic field and with relatively strong interactions, additional off-resonant state-changing dipolar interaction terms (Δ*ℓ* = ± 2, which do not conserve the net internal angular momentum nor the individual state populations), also have a small influence on the state population dynamics^49^. The full interaction Hamiltonian is

2 $${H}_{{{{{{{{\rm{int}}}}}}}}}=\mathop{\sum}\limits_{m,n}\mathop{\sum}\limits_{i,j,{i}^{{\prime} },{j}^{{\prime} }}{V}_{ij{i}^{{\prime} }{j}^{{\prime} }}^{mn}{e}^{i{\Delta }_{ij}^{{i}^{{\prime} }{j}^{{\prime} }}t}{\hat{c}}_{i,m}^{{{{\dagger}}} }{\hat{c}}_{j,m}{\hat{c}}_{{j}^{{\prime} },n}^{{{{\dagger}}} }{\hat{c}}_{{i}^{{\prime} },n}+{{{{{{{\rm{h}}}}}}}}.{{{{{{{\rm{c}}}}}}}}.\,,$$

where ${V}_{ij{i}^{{\prime} }{j}^{{\prime} }}^{mn}=\langle {j}_{m}{i}_{n}^{{\prime} }| {\hat{V}}_{{{{{{{{\rm{dd}}}}}}}}}| {i}_{m}{j}_{n}^{{\prime} }\rangle$ with the dipolar interaction operator ${\hat{V}}_{{{{{{{{\rm{dd}}}}}}}}}=\frac{1}{4\pi {\epsilon }_{0}{r}_{mn}^{3}}[\frac{1}{2}(2{\hat{d}}_{m}^{0}{\hat{d}}_{n}^{0}+{\hat{d}}_{m}^{+}{\hat{d}}_{n}^{-}+{\hat{d}}_{m}^{-}{\hat{d}}_{n}^{+})-\frac{3}{2}({\hat{d}}_{m}^{-}{\hat{d}}_{n}^{-}+{\hat{d}}_{m}^{+}{\hat{d}}_{n}^{+})]$ between atom *m* and atom *n* (${\hat{d}}^{0}$, ${\hat{d}}^{+}$, and ${\hat{d}}^{-}$ are the respective dipole moment operators for *π*, *σ*^+^ and *σ*^−^ transitions), and where ${\Delta }_{ij}^{{i}^{{\prime} }{j}^{{\prime} }}$ is the energy difference between the two-body state configurations ${\vert i\rangle }_{m}{\vert \,\, {j}^{{\prime} }\rangle }_{n}$ and ${\vert \,\, j\rangle }_{m}{\vert {i}^{{\prime} }\rangle }_{n}$. Here, the state indices $i,j\,({i}^{{\prime} },{j}^{{\prime} })$ also cover other Rydberg sublevels in both 42*P* manifolds to account for strong non-resonant dipolar interactions.

The dipolar interactions enrich the synthetic lattice dynamics by correlating the Rydberg electron “motion” on different atoms. For two neighboring atoms starting in the state ${\vert i\rangle }_{m}{\vert i\rangle }_{n}$, symmetric single-particle hopping to the state ${\vert+\rangle }_{ij}=({\vert i\rangle }_{m}{\vert \,\, j\rangle }_{n}+{\vert \,\, j\rangle }_{m}{\vert i\rangle }_{n})/\sqrt{2}$ is driven off-resonance by the presence of interactions, as ${\left\vert+\right\rangle }_{ij}$ is shifted in energy by *V*_*ij*_. When the interactions are sufficiently strong (∣*V*_*ij*_ ≫ Ω/2∣), interactions will suppress uncorrelated hopping processes, somewhat analogous to how on-site interactions suppress uncorrelated atom hopping in optical lattices^54^. For pairs, transport can still occur by pair-hopping across individual lattice links at a rate ${V}_{ii\to jj} \, \approx -{\Omega }^{2}{e}^{i2{\phi }_{ij}}/(2{V}_{ij})$^49^. Three things to note about transport in the *V* ≫ Ω limit: (1) interactions will considerably slow down the dynamics of pairs, (2) bound pairs should experience twice the tunneling phase (flux for closed paths) as experienced by single particles, and (3) the matrix elements for resonant three-atom (and higher) hopping processes are even further reduced.

These interactions have been predicted to induce emergent quantum strings and membranes in the ground state^26–28^, relating to self-trapped^55^ multi-atom bound states in 1D and 2D atom arrays. Ground state strings and membranes, self-bound by interactions, are nonetheless predicted to be delocalized in the synthetic dimension for uniform, translationally invariant interactions (*V*_*i*,*i*+1_ = *V*). In this work and for generic state arrangements, however, the *V*_*ij*_ terms have significant structure across the synthetic lattice and can be considered as a kind of interaction disorder^56^, which may reasonably be expected to localize dipolar bound states.

For interacting pairs, we restrict ourselves to measuring the population of $\left\vert 0\right\rangle$ for each atom, as the basis rotation pulses used for the readout of other internal states are influenced by the presence of strong interactions. Figure 2d shows the average probability for a pair of atoms to reside at the site $\left\vert 0\right\rangle$. We compare the data to no-free-parameter simulations that incorporate the single-particle dynamics of Eq. (1) as well as all interaction effects of Eq. (2). The theory lines and confidence intervals are based on the calibrated parameters and interaction values, as well as their uncertainties, and further account for the independently determined Rydberg state preparation infidelity, which leads to ~15% of the “pair data” cases consisting of just a single Rydberg-excited atom^49^. For pairs in the intermediate interaction regime [*V*/Ω = 1.8(1)], we observe that the dipolar interactions strongly modify the dynamics, in general slowing down the dynamics and decreasing the amplitude of recurrences. As a more direct probe of interaction-driven correlations, we measure the two-atom correlator ${{{{{{{{\mathcal{C}}}}}}}}}_{00}=\langle {\hat{c}}_{0,L}^{{{{\dagger}}} }{\hat{c}}_{0,L}{\hat{c}}_{0,R}^{{{{\dagger}}} }{\hat{c}}_{0,R}\rangle -\langle {\hat{c}}_{0,L}^{{{{\dagger}}} }{\hat{c}}_{0,L}\rangle \langle {\hat{c}}_{0,R}^{{{{\dagger}}} }{\hat{c}}_{0,R}\rangle$ with *L* and *R* referring to the left and right atoms of isolated pairs. This quantity vanishes in the absence of interactions, and grows as the atoms develop correlations of their positions in the synthetic lattice. Both the *P*_0_ and *C*_00_ dynamics are in fairly good agreement with our textbook theory expectations, confirming that dipolar Rydberg atom arrays are a promising platform for exploring coherent interactions in tunable synthetic lattices.

**Fig. 3:**
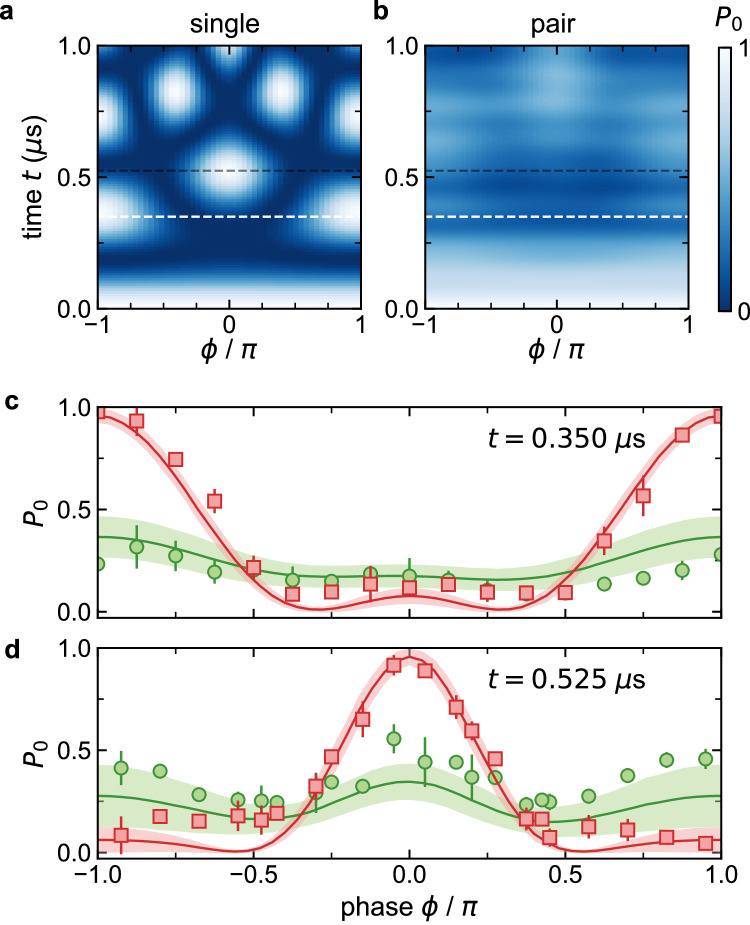
Flux-dependence of atom and atom pair dynamics. **a** Numerically calculated plot of the average probability vs. time *t* and flux *ϕ* for atoms initialized at state $\left\vert 0\right\rangle$ to remain at that state (*P*_0_). **b** The same quantity as plotted in (**a**), and with a common color bar at right, but as calculated for interacting pairs of atoms, for *V*/Ω = 1.8. **c**, **d** Measured (SPAM-corrected^49^) *P*_0_ for single atoms (red squares) and pairs (green circles) after evolution times of *t* = 0.350 *μ*s and 0.525 *μ*s, corresponding to cuts along the white and black dashed lines of panels (**a**, **b**). The typical singles dataset is derived from ~200 post-selected images while pairs relate to ~50 post-selected images. The theory lines and shaded regions are as in Fig. 2, accounting for dynamics according to Eq. (1) and Eq. (2) as well as calibrated parameter values and known uncertainties^49^, as described in the text. The characteristic hopping and interaction terms are Ω/*h* = 1.92(6) MHz and *V*/*h* = 3.44(8) MHz. Error bars in (**c**, **d**) are the standard error of multiple independent data sets.

We now more thoroughly explore in Fig. 3 the flux-dependent dynamics for individual atoms and atom pairs. Figure 3a, b shows numerical simulations of the full flux-dependence of the *P*_0_ dynamics for singles and pairs (with the simulations incorporating all the same elements as in Fig. 2). For singles, as described before, the changing timescales for recurrences of the measure *P*_0_ simply reflect the flux-modified gaps of the system’s energy spectrum. For our measurements, we probe precisely at the first expected *P*_0_ recurrence time for singles at flux values of *ϕ* = *π* and 0, namely at *t* = 0.350 *μ*s in Fig. 3c and *t* = 0.525 *μ*s in Fig. 3d, respectively. For singles, we observe good general agreement with the full flux-dependence of the expected *P*_0_ dynamics. For doubles, we observe both in theory and experiment that *P*_0_ remains relatively small and has low contrast as a function of *ϕ*, owing to the dynamics being slowed down relative to singles. We note that the apparent *π*-periodicity of the doubles response after 0.525 *μ*s is somewhat suggestive of the expected higher flux sensitivity of pairs^49^.

### Dynamics in multi-atom arrays

**Fig. 4:**
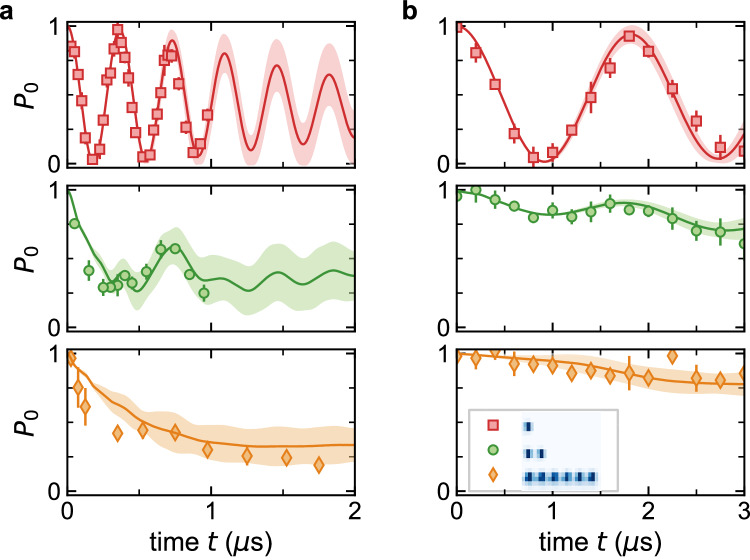
Scrambling and self-trapping in few-atom arrays. **a** Dynamics for single atoms (red squares), atom pairs (green circles), and six-atom arrays (orange diamonds) under a flux of 1.00(2)*π* and for a characteristic interaction-to-hopping ratio of *V*/Ω = 1.8(1) with Ω/*h* = 1.92(6) MHz. The singles and pairs data are the same as for Fig. 2c, f. **b** Same quantities as in (**a**), but for reduced hopping amplitude Ω/*h* = 0.38(1) MHz and *V*/Ω = 9.0(5). All data are SPAM-corrected^49^. Experimental error bars are the standard error from multiple independent data sets. The theory lines and shaded regions account for known parameter uncertainties, along with influences of Rydberg excitation inefficiencies and other experimental noise sources^49^.

Finally, we explore how interactions in Rydberg synthetic dimensions can have an even richer influence on dynamics as we extend towards many-atom arrays. In Fig. 4a, b, we contrast the *ϕ* = 1.00(2)*π* dynamics of one, two, and six-atom arrays for intermediate [(a), *V*/Ω = 1.8(1), Ω/*h* = 1.92(6) MHz] and large [(b), *V*/Ω = 9.0(5), Ω/*h* = 0.38(1) MHz] interaction-to-tunneling ratios. For both cases, *P*_0_ oscillates with high coherence and a single frequency for single atoms (there is only a single energy gap value of Eq. (1) at *π* flux). However, interactions lead to qualitatively different dynamics in multi-atom arrays^49^. In Fig. 4a, for *V*/Ω ~ 1.8, the macroscopic observable *P*_0_ shows coherent revivals with a structured time-dependence for pairs, and less oscillations but a clear decay for six-atom clusters. Specifically, numerical simulations for the six-atom array show a rapid relaxation to *P*_0_ ≈ 1/4, suggestive of an approach to ergodicity in this closed many-body system.

The dynamics of arrays relative to singles changes remarkably for strong interactions, *V*/Ω ≈ 9, as shown in Fig. 4b. For pairs, we observe only a very slow decay of *P*_0_ over the 3 *μ*s measurement window, consistent with the prediction of pair-hopping^57^ being slowed down (by a factor of 9) relative to singles in this large *V*/Ω limit. The *P*_0_ dynamics is still further reduced for the six-atom clusters. Indeed, interaction-driven self-trapping is expected in this strong interaction regime, and the resulting states can be considered simpler excited-state analogs of ground-state quantum strings^26–28^. To note, when *V*/Ω ≫ 1, one should also expect the system to be prone to Hilbert space fragmentation^58,59^ and fully arrested dynamics under added perturbations (e.g., gradients or disorder).

## Discussion

These observations of interaction-driven self-immobilization pave the way for future explorations of ground state quantum strings and membrane phases in Rydberg arrays^26–28^, along with more exotic localized phases that may arise due to structured, inhomogeneous interactions^56^. More generally, the coherent dynamics and clear interaction effects observed in this few-state, few-atom study bodes well for extensions to complex internal-state lattices composed of dozens of Rydberg states, as well as to larger 1D and 2D real-space atom arrays. This synthetic dimensions system based on arrays of dipolar spins^26^, while having some peculiar features (e.g., one “excitation” per real-space location, challenging multi-state readout), promises to be a unique playground for exploring the influence of strong interparticle interactions on topological and localization phenomena. Moreover, this system offers a powerful new approach to the study of many-body nonequilibrium dynamics.

## Methods

### Preparation of the atom arrays

Our experiments are based on commonly used techniques for the trapping and lossless imaging of atoms in optical tweezers^43^. We load ^39^K atoms into one-dimensional optical tweezer arrays generated by diffraction of 780 nm laser light from an acousto-optic deflector (AA Opto-Electronic part number DTSX-400-780). For the measurements in this paper, we use two different tweezer array patterns: the pattern of seven two-site dimers depicted in Fig. 1a, b as well as a pattern of three six-site clusters used for the data in Fig. 4.

After probabilistic loading of the traps, we perform nondestructive fluorescence imaging to determine the tweezer occupations for subsequent post-selection of the data. Our imaging of ground state atoms is mostly lossless (>99% survival probability) and provides good discrimination (>99%) between occupied and unoccupied tweezers, as detailed in ref. ^45^.

Prior to free-space release of the atoms and their excitation to Rydberg states, the atoms are cooled by gray molasses cooling, optically pumped to a stretched hyperfine level $\left\vert F,{m}_{F}\right\rangle=\left\vert 2,2\right\rangle$, and further cooled to a final temperature of ~4 *μ*K by adiabatic decompression of the optical tweezer trapping potential^49^.

### Microwave control of the synthetic Rydberg-state lattice

The optically pumped ^39^K ground state atoms are transferred to the Rydberg level $\vert 42{S}_{1/2},{m}_{J}=1/2\rangle$ by stimulated Raman adiabatic passage (STIRAP), as detailed in the Supplement^49^. Following this Rydberg state initialization, we form the “synthetic Rydberg-state lattice” by applying four phase-stable microwave tones that coherently couple the microwave levels $\left\vert 0\right\rangle$, $\left\vert 1\right\rangle$, $\left\vert 2\right\rangle$, and $\left\vert 3\right\rangle$. The resonance frequencies are calibrated by Rabi and Ramsey spectroscopy, the individual Rabi rates are calibrated by the measurement of state-to-state Rabi oscillations, and the global flux of the four-state diamond configuration is calibrated based on the Rydberg state population dynamics of individual atoms.

### Correction of state preparation and measurement (SPAM) errors

The bare measurements of the individual state populations are slightly different from the data presented in Figs. 1–4, which are “corrected” to account for state preparation and measurement (SPAM) errors. A full discussion of the SPAM correction is presented in the Supplementary Materials^49^.

There are several effects that limit our state preparation and measurements. For the initial and final detection of ground state atoms, the actual survival probability and occupation discrimination (between occupancy and an empty tweezer) are quite high (>99%)^45^, and their impact is excluded from the SPAM correction. Faithful excitation of the $\vert 0\rangle \equiv \vert 42{S}_{1/2},{m}_{J}=1/2\rangle$ Rydberg state is limited by both optical pumping inefficiency and imperfect STIRAP transfer between the pumped state ($\left\vert F,{m}_{F}\right\rangle=\left\vert 2,2\right\rangle$) and the $\left\vert 0\right\rangle$ Rydberg state. Prior to Rydberg excitation, the atoms are optically pumped to the $\left\vert F,{m}_{F}\right\rangle=\left\vert 2,2\right\rangle$ state with 98(1)% efficiency^49^. Prior to turning on the microwaves that introduce the synthetic lattice, the atoms are excited to the Rydberg level $\left\vert 0\right\rangle$ via stimulated Raman adiabatic passage (STIRAP) with a one-way efficiency of 94(1)%. Thus, the typical state preparation fidelity is 92%. Also accounting for loss during release-and-recapture (because the tweezers are extinguished while the atoms are excited to Rydberg levels), we measure an overall “upper baseline” of *P*_*u*_ = 0.88(1) from the combination of state preparation errors and atom loss.

We are also limited in terms of detection discrimination between the chosen (intentionally de-excited) Rydberg level and the other Rydberg levels, which experience spontaneous emission decay that results in their recapture and spurious detection as ground-state atoms. This leads to an infidelity of discrimination between the intentionally de-excited Rydberg state and the other Rydberg levels. We experimentally measure a “lower baseline” of *P*_*l*_ = 0.21(1), which is predominantly due to the decay, recapture, and detection of the short-lived *n* = 42 Rydberg states.

To correct for these known errors, we renormalize the measured average state populations in terms of the directly measured (bare) average state populations, *P*_*i*_, as ${P}_{i}=({P}_{i}^{{{{{{{{\rm{bare}}}}}}}}}-{P}_{l})/({P}_{u}-{P}_{l})$. To note, for the states $\left\vert 1\right\rangle$, $\left\vert 2\right\rangle$, and $\left\vert 3\right\rangle$, we do not further account for any errors associated with the microwave pulses applied prior to Rydberg state de-excitation.

### Supplementary information

Supplementary Information

Peer Review File

## Data Availability

All of the experimental data from this work are deposited in the Zenodo database under accession code 10.5281/zenodo.10797815.
