# Supplementary material for: Strongly interacting Rydberg atoms in synthetic dimensions with a magnetic flux
Source: Nat Commun. 2024 Mar 27;15:2675. doi: 10.1038/s41467-024-46823-6 (PMC10973509; doi:10.1038/s41467-024-46823-6)
Supplement: Supplementary file 1 — Supplementary Information [file 41467_2024_46823_MOESM1_ESM.pdf]

Supplemental Material for
"Strongly interacting Rydberg atoms in synthetic dimensions with a magnetic flux"

Tao Chen,[1,*] Chenxi Huang,[1,*] Ivan Velkovsky,[1]
Kaden R. A. Hazzard,[2,3,4,†] Jacob P. Covey,[1,‡] and Bryce Gadway[1,§]

[1]*Department of Physics, University of Illinois at Urbana-Champaign, Urbana, IL 61801-3080, USA*
[2]*Department of Physics and Astronomy, Rice University, Houston, TX 77005, USA*
[3]*Rice Center for Quantum Materials, Rice University, Houston, TX 77005, USA*
[4]*Department of Physics, University of California, Davis, CA 95616, USA*

Experimental initialization procedure

We begin our experiments by loading ^{39}K atoms into one-dimensional optical tweezer arrays generated by diffraction of 780 nm laser light from an acousto-optic deflector (AA Opto-Electronic part number DTSX-400-780). Every cycle of the experiment (having a duration of 1.3 s), atoms are first probabilistically loaded into the tweezer traps with an

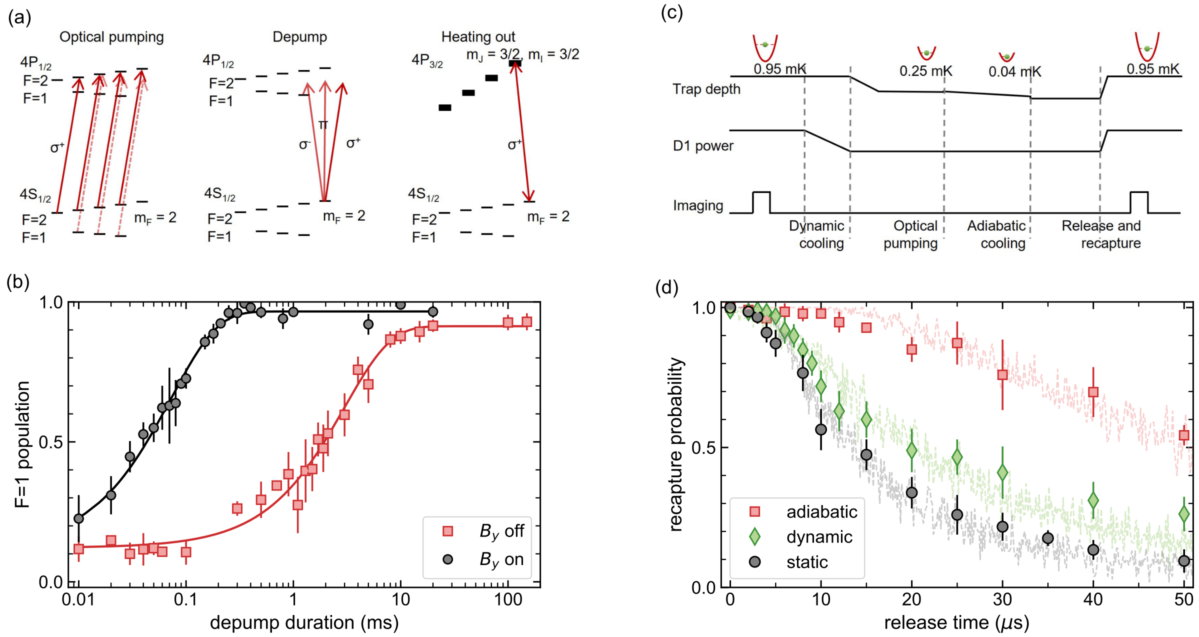

FIG. S1. **Initial state preparation and temperature optimization procedure.** **(a)** Depump-heating out sequence to optimize the σ^+ polarization of the D1 optical pumping beam. After pumping atoms into $|F=2, m_F=2\rangle$ dark state, we turn the repump component off, and the atoms get depumped into $|F=1\rangle$ states by the imperfect π and σ^- light in the optical pumping beam. Then we apply a heating beam (weak D2 light resonant to the $|F=2, m_F=2\rangle \leftrightarrow |4P_{3/2}, m_j=3/2\rangle$ cycling transition) to remove $|F=2\rangle$ atoms. Measurement of the surviving atom number provides a measurement of the $|F=1\rangle$ population after the depumping step. **(b)** Measured $F=1$ population versus the depump duration for the case with only magnetic field along the z-direction (red square, B_y off) and that with an additional magnetic field in the y-direction (black circle, B_y on). Since B_y changes the quantization axis, the atoms suffer from a relatively rapid depumping process. The exponential fittings (solid lines) give the corresponding time constant: $\tau_{\rm op} = 72(2)$ μs with B_y on and $\tau_{\rm dp} = 3.0(2)$ ms with B_y off. **(c)** Time sequence for optimization of the atom temperature with release-recapture measurement: (1) After first imaging with a trap depth of ~0.95 mK, we dynamically ramp down the D1 power at a fixed trap depth, cooling the atoms from ~60 μK (static cooling without changing D1 power) down to ~20 μK; (2) Then, we ramp the trap depth down to ~0.25 mK and perform optical pumping with D1 frequency shifted to compensate the revised trap light shift; (3) We further adiabatically ramp the trap depth down to ~40 μK to further cool the atoms to ~4 μK. **(d)** Release and recapture measurements of the atom temperature after different cooling processes. The temperatures are resolved by fitting the respective experimental data sets to Monte Carlo simulations (dashed lines).

average probability of ∼55%. Two different tweezer patterns are used for the studies presented: the pattern of seven two-site dimers depicted in Figs. 1a,b as well as a pattern of three six-site clusters used for the data in Fig. 4.

After an initial loading, the samples of atoms are nondestructively imaged a first time for subsequent post-selection. The fluorescence imaging, with a duration of 40 ms, is characterized by a survival probability >99% and a discrimination fidelity (for the assignment of an atom occupancy or vacancy) >99% [1]. The atoms are then re-cooled by gray molasses [1, 2] as well as adiabatic trap decompression (lowering the depth of our Gaussian tweezer traps from an initial depth of ∼0.95 mK to a final depth of 40 μK) to a final temperature of ∼4 μK as calibrated by release and recapture [3]. This overall preparation procedure, as well as the release-and-recapture probability curves (and associated numerical comparisons for temperature estimation) can be seen in Fig. S1c,d. The three release-and-recapture curves in Fig. S1d are respectively characterized by a temperature of ∼60 μK (black data points and theory) under static D1 cooling in the trap at its initial depth, a reduced temperature of ∼20 μK (green data points and theory) following a dynamical reduction of the D1 molasses cooling power, and a minimum temperature of ∼4 μK (red data points and theory) following an adiabatic ramp down of the optical tweezer trap to a final depth of ∼40 μK.

In preparation for the Rydberg studies, prior to the final adiabatic cooling stage, the trapped atoms are optically pumped to a single ground internal state, $|4S_{1/2}, F = 2, m_F = 2\rangle$, with ∼98(1)% efficiency. As depicted in Fig. S1a,b, the efficiency is estimated by comparing the characteristic depumping time for the case of the actual bias field $B_z = 27$ G implemented in experiment ($\tau_{\mathrm{dp}} = 3.0(2)$ ms, B_y off) to the case where an additional magnetic field is added along the y axis ($\tau_{\mathrm{op}} = 72(2)$ μs, B_y on, with $B_y \sim 12$ G). This additional field B_y disrupts the polarization purity of the depumping beam relative to the total quantization axis, and the corresponding measurement provides a lower estimate for the depumping rate for unpolarized light. The depumping times for these two situations can be combined to estimate the optical pumping (OP) efficiency $\eta = 1 - \tau_{\mathrm{op}}/\tau_{\mathrm{dp}} = 98(1)\%$ [4].

The excitation of the atoms to Rydberg levels (principal quantum number $n = 42$) is performed after releasing the atoms from the optical tweezer traps, which are weakly anti-trapping (with a polarizability that is roughly 30 times lower in magnitude as compared to that for the ground state) for the target Rydberg level. After ∼0.2 μs of release, a two-photon STIRAP pulse is applied, as depicted in Fig. S2. This excitation involves laser light slightly (∼15 MHz) detuned from the $|4S_{1/2}, F = 2, m_F = 2\rangle \leftrightarrow |5P_{1/2}, F = 2, m_F = 1/2\rangle$ transition ("lower leg," having a wavelength of ∼405 nm) and the $|5P_{1/2}, F = 2, m_F = 1/2\rangle \leftrightarrow |42S_{1/2}, m_J = 1/2\rangle$ transition ("upper leg," having a wavelength of ∼975 nm). The two lasers used for STIRAP are stabilized via Pound-Drever-Hall (PDH) locking to a common ultra-low expansion (ULE) optical cavity (Stable Laser Systems). Peak single-photon (resonant) Rabi rates of $\sim 2\pi \times 20$ MHz for the lower and upper legs of the STIRAP transition are achieved by focusing the combined

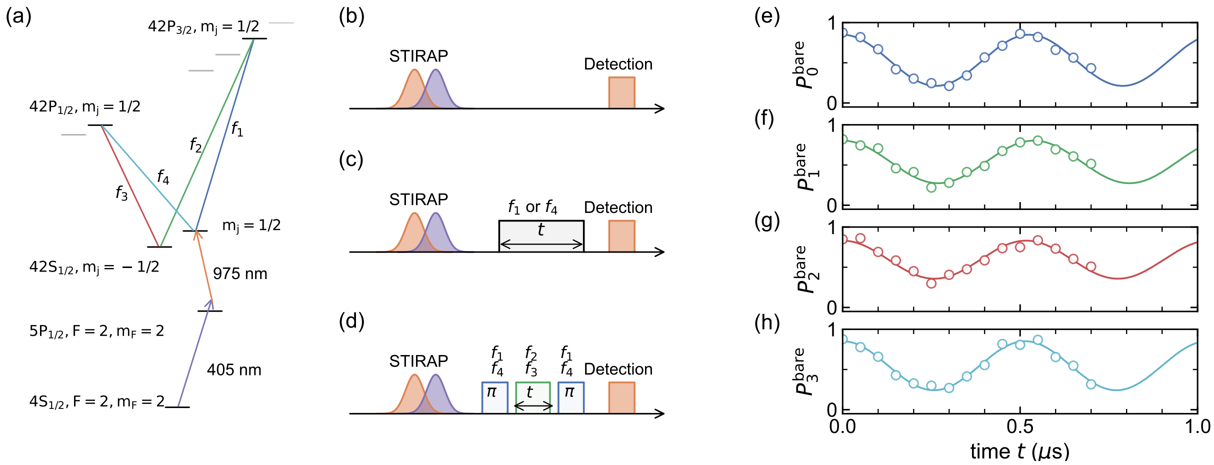

FIG. S2. **Calibration of the Rabi frequencies between each Rydberg state pair in isolated single atoms.** **(a)** Level structure used in our experiment. **(b)** Time sequence with only STIRAP pulses and 975 nm de-excitation pulse to calibrate the baselines of the oscillation between Rydberg state pairs. **(c)** Time sequence with an extra MW pulse to measure the Rabi oscillation between $|0\rangle = |42S_{1/2}, m_j = 1/2\rangle$ and $|1\rangle = |42P_{3/2}, m_j = 1/2\rangle$ ($|3\rangle = |42P_{1/2}, m_j = 1/2\rangle$) driven by f_1 (f_4). **(d)** Time sequence with two extra π-pulses to measure the Rabi oscillation between $|2\rangle = |42S_{1/2}, m_j = -1/2\rangle$ and $|1\rangle = |42P_{3/2}, m_j = 1/2\rangle$ ($|3\rangle = |42P_{1/2}, m_j = 1/2\rangle$) driven by f_2 (f_3). **(e-h)** Measured uncorrected state population dynamics when driving pairwise Rabi oscillations between states addressed by the tones f_1 to f_4, respectively (from top to bottom). The solid line fits are damped sine functions used to calibrate the respective Rabi rates we use as $\{\Omega_{01}, \Omega_{12}, \Omega_{23}, \Omega_{30}\}/h = \{1.90(3), 1.86(4), 1.93(4), 1.94(4)\}$ MHz.

laser beams to respective waists of 40 μm and 30 μm. The peak powers $I_{0,L/U}$ at the atoms are roughly 3 mW for the "lower leg" and 300 mW (after amplification by a tapered amplifier) for the "upper leg," respectively. We use Gaussian-shaped pulses for both the lower and upper legs, as shown in Fig. S2b, the intensities of which follow the formula $I_{L/U}(t) = I_{0,L/U}\exp\left[-\frac{(t\pm\Delta t/2)^2}{\sigma^2}\right]$ with $\Delta t = 0.3$ μs and $\sigma = 0.3$ μs. Under these conditions, we achieve a one-way STIRAP efficiency of $\sim$94(1) %.

The synthetic lattice – state configuration and calibration

The mapping between bare Rydberg levels and the sites of our synthetic lattice are detailed in the main text Figs. 1c,e. These chosen assignments are informed by a few simple considerations: (i) we would like all of the state-to-state transitions to be achieved by dipole-allowed first-order processes, (ii) we desire for the resonant exchange interactions to only occur between nearest neighbors in the synthetic dimension, and (iii) for technical considerations, we require that all of the transitions can be addressed with only a moderate microwave bandwidth. The "folded diamond" layout of Fig. S2a satisfies these design goals.

The microwave transition frequencies between each Rydberg state pair in our system lie in the vicinity of 48 GHz, as shown in Fig. S2a. We first input a single-tone microwave signal at $\sim$12 GHz (generated from a Vaunix Lab Brick device) into a 4× frequency multiplier (Marki AQA-2156). We then mix this high-frequency carrier signal with two multi-tone arbitrary waveform signals (generated from a Teledyne SDR14TX card) via an IQ frequency mixer (Marki MMIQ-4067L) to produce the required sidebands to resonantly couple the relevant pairs of states in our system.

Readout from the various Rydberg levels is achieved through combinations of microwave state-swapping pulses and optical depumping on the "upper leg" 975 nm transition (*i.e.*, applying only the "upper leg" of the STIRAP transitions to depopulate the $|0\rangle$ Rydberg state to the atomic ground states, through the short-lived $5P$ levels). For the $|0\rangle$ state that we initially populate via STIRAP, the readout for measuring the population P_0 simply involves applying near-resonant depumping on the upper leg 975 nm transition (followed by ground state imaging). To note, depumping from the nearby $|2\rangle$ state (75 MHz away in energy at these moderate bias fields) is avoided by using sufficiently weak intensities of the 975 nm depumping light. As described in the main text, the ability to depump both the $|0\rangle$ and $|2\rangle$ state simultaneously by applying high-intensity depump light provides a useful way to measure

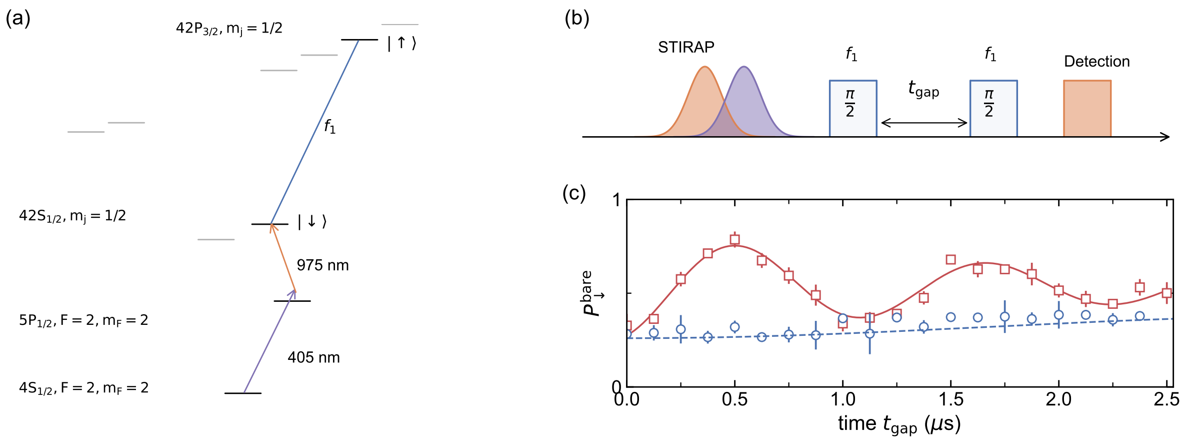

FIG. S3. **Calibration of the dipole-dipole interaction strength. (a)** Level structure used to measure the dipolar exchange interaction with $|{\downarrow}\rangle = |0\rangle = |42S_{1/2}, m_j = 1/2\rangle$ and $|{\uparrow}\rangle = |1\rangle = |42P_{3/2}, m_j = 1/2\rangle$. The interatomic distance is set to ≈ 10 μm, at exactly twice the spacing as used for the experiments described in the main text. **(b)** Timing diagram of the sequence used to measure Ramsey coherence oscillations for pairs of interacting atoms. After initially preparing a pair of atoms in $|{\downarrow\downarrow}\rangle$ via STIRAP, a strong microwave $\pi/2$ pulse (Rabi frequency $\sim 2\pi \times 4.0$ MHz) is applied to rotate the atom pair to a product state $|{\downarrow} + i{\uparrow}\rangle|{\downarrow} + i{\uparrow}\rangle/2 = [|{\downarrow\downarrow}\rangle - |{\uparrow\uparrow}\rangle + i(|{\uparrow\downarrow}\rangle + |{\downarrow\uparrow}\rangle)]/2$. Then we let the system freely evolve for a duration of t_{gap}, during which the time evolution follows $[|{\downarrow\downarrow}\rangle - |{\uparrow\uparrow}\rangle + ie^{iV_{01}^{\text{cal}}t_{\text{gap}}/\hbar}(|{\uparrow\downarrow}\rangle + |{\downarrow\uparrow}\rangle)]/2$ as the state $|{\uparrow\downarrow}\rangle + |{\downarrow\uparrow}\rangle$ has an eigenenergy of V_{01}^{cal}. We finally read out the population in $|{\downarrow}\rangle$ after applying an identical microwave $\pi/2$ pulse and 975 nm de-excitation pulse. **(c)** Detected population in $|{\downarrow}\rangle$ state versus the free evolution time t_{gap} for non-interacting single atoms (blue circles) and for the atoms of interacting pairs (red squares). The fitting (solid line) to data for atom pairs with a damped sine function gives the oscillation frequency, *i.e.*, the interaction strength, $V_{01}^{\text{cal}}/h = 0.86(2)$ MHz. In contrast, fitting (dashed line) to the single-atom data with a sine function shows only a slow variation, indicating the frequency of microwave f_1 is detuned from resonance by $\sim$150 kHz.

the $|2\rangle$ state population as $P_2 = P_{0+2} - P_0$. For the states $|1\rangle$ and $|3\rangle$, relating to $42P$ levels, the populations P_1 and P_3 can be read out by applying π pulses on the $|0\rangle \leftrightarrow |1\rangle$ or the $|0\rangle \leftrightarrow |3\rangle$ transition prior to measurement of the $|0\rangle$ state population. And, while not utilized in this study, coherences between the sites in the synthetic dimension can also be read out in such a way.

Figure S2 details the procedure for calibrating the effective tunneling rates (transition Rabi rates) along the various links of the lattice. Because the various transitions involve different sets of states and require different polarizations of microwaves, the individual amplitudes of the tones f_{1-4} are adjusted to achieve a uniform tunneling rate across all links. As described in the text, the overall flux ϕ of the synthetic diamond lattice is calibrated based on the dynamical response of isolated single atoms. Specifically, it is based upon fitting a Gaussian to the response of the singles data in Fig. **??**d, after an evolution time of 0.525 μs that corresponds to roughly $t = h/\Omega$. The value of ϕ is set by controlling the source phase of the f_1 frequency tone relative to the other tones. To note, we find in experiment that the flux ϕ is extremely stable, with no noticeable variations on the week-long timescales hitherto explored.

Corrections for preparation and readout infidelity

The primary data we measure for all state populations P_{0-3} appear similar to those presented in Fig. S2 and Fig. S3. There are two limiting quantities to note. First, there is an upper baseline value that is on average equal to $P_u = 0.88(1)$, which stems from inefficiencies of STIRAP and release-and-recapture survival. There is also a lower baseline of the measurements, having a value $P_l = 0.21(1)$, that we believe stems from the decay (and subsequent recapture) of the short-lived $n = 42$ Rydberg states. This lower baseline represents a lack of fidelity in discriminating atoms from being successfully depumped from the state of choice as opposed to decaying from any of the Rydberg levels. These infidelities limit the contrast of single atom dynamics, and more importantly limit our ability to faithfully measure atom-atom correlation dynamics.

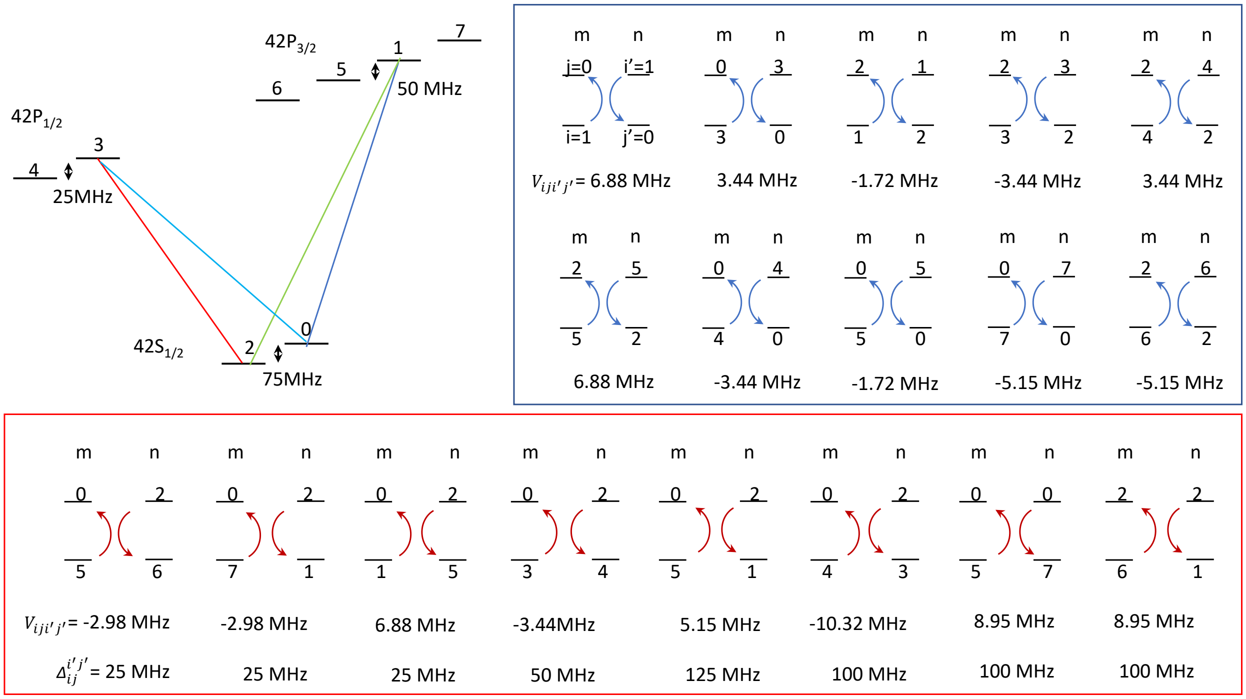

FIG. S4. **All dipole-dipole interaction terms relevant to our experimental scheme.** Blue box: Resonant flip-flop interactions with $\Delta_{ij}^{i'j'} = 0$. We list the interaction energies (reduced by Planck's constant) in MHz. Red box: Non-resonant state-changing interaction terms. The listed interaction strength $V_{iji'j'}$ values (also reduced by Planck's constant and listed in MHz) are based on the experimentally calibrated $V_{01} = 6.88$ MHz scaled by the respective C_3 coefficients calculated with the Alkali Rydberg calculator (ARC) package [5]. We also list the configurational detuning $\Delta_{ij}^{i'j'}$ with respect to our synthetic lattice states for each of the processes (also in MHz).

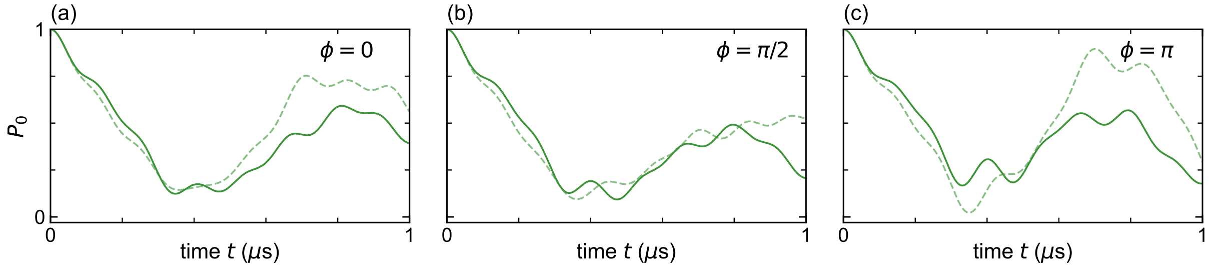

FIG. S5. **The role of non-resonant and state non-conserving dipolar interactions.** (a-c) Simulation results of the P_0 dynamics for Rydberg pairs under different flux values, $\phi = 0$, $\pi/2$, and π. We show simulations both for the full set of dipolar exchange interaction terms (solid lines) as well as the case of only the resonant, state-conserving flip-flop interactions (dashed lines).

For all of the data in the paper, we "correct" for these known infidelities in the following way: we define the corrected populations P_i in relation to the measured bare populations P_i^{bare} as $P_i = (P_i^{\text{bare}} - P_l)/(P_u - P_l)$.

Dipolar interactions – theoretical expectation and experimental calibration

In the language of our tight-binding "synthetic lattice" model, the dipole-dipole interaction Hamiltonian is

$$H_{\text{int}} = \sum_{m,n} \sum_{i,j,i',j'} V_{iji'j'}^{mn} e^{i\Delta_{ij}^{i'j'} t} \hat{c}_{i,m}^\dagger \hat{c}_{j,m} \hat{c}_{j',n}^\dagger \hat{c}_{i',n} + \text{h.c.} \ , \tag{S1}$$

where $V_{iji'j'}^{mn} = \langle j_m i'_n | V_{\text{dd}} | i_m j'_n \rangle$ with the dipolar interaction operator $V_{\text{dd}} = \frac{1}{4\pi\epsilon_0 R_{mn}^3} \left[\frac{1}{2}(2d_m^0 d_n^0 + d_m^+ d_n^- + d_m^- d_n^+) - \frac{3}{2}(d_m^- d_n^- + d_m^+ d_n^+) \right]$ between atom m and atom n (d^0, d^+ and d^- are the respective dipole moment operators for π, σ^+ and σ^- transitions), and where $\Delta_{ij}^{i'j'}$ is the energy difference between $|i_m\rangle \leftrightarrow |j_m\rangle$ and $|i'_n\rangle \leftrightarrow |j'_n\rangle$ transitions (or, equivalently, the energy difference between the two-body state configurations $|i_m\rangle|j'_n\rangle$ and $|j_m\rangle|i'_n\rangle$). Here the state index i, j (i', j') also covers other unused sublevels in both $42P$ manifolds, as displayed in Fig. S4, to include the strong state-changing dipolar interactions. To note, the form of Eq. S1 and the corresponding main text form both neglect the azimuthal phase contribution of the $\Delta\ell = \pm 2$ interaction terms.

There are four primary dipolar exchange processes that we care about (*i.e.*, they are the only four processes that are resonant for the states intentionally populated in our experiment), occurring between pairs of atoms occupying the states $|0\rangle$ and $|1\rangle$ (with an energy scale V_{01}), $|1\rangle$ and $|2\rangle$ (V_{12}), $|2\rangle$ and $|3\rangle$ (V_{23}), and $|3\rangle$ and $|0\rangle$ (V_{30}). Based on the construction of our synthetic lattice, all of these resonant "flip-flop" terms occur between pairs of atoms residing on neighboring sites of our synthetic lattice. Importantly, in this work the population dynamics is also impacted by the presence of relatively strong state-changing dipolar interactions that are not very far off from resonance (because we operate with only a moderate quantization field). The full enumeration of resonant population-conserving ($\Delta\ell = 0$) and off-resonant population non-conserving ($\Delta\ell = \pm 2$) dipolar interaction terms are presented in Fig. S4. The resonant terms are listed in the blue box, and all relevant (not off-resonant by more than 125 MHz) non-resonant exchange terms are enumerated in the red box.

For the plots in the main text, all interaction terms as well as known sources of parameter uncertainty and variation are incorporated in the theory curves (and confidence intervals). In Fig. S5, to help elucidate the role that the off-resonant dipolar interactions play, we plot simulations of the ideal (no SPAM and no parameter uncertainties) pair-atom dynamics for the case of the full set of expected dipolar interaction terms (solid lines) as well as the situation in which only the state-conserving, resonant interactions contribute (dashed lines). As can be seen, these slightly "non-ideal" terms contribute to the dynamics at longer times. Looking forward, such terms can be effectively eradicated by working at larger bias magnetic fields or with smaller values of the interaction strengths (or by working with the quantization field aligned along the array).

Based on our imaged tweezer patterns and the designed magnification of our imaging system, we expect our tweezer trap spacing to be $\sim$5 μm. Based on this spacing and the known [5] C_3 coefficients for the Rydberg states we consider, we can obtain estimates for these various dipolar interaction energies. Because the imaging system's

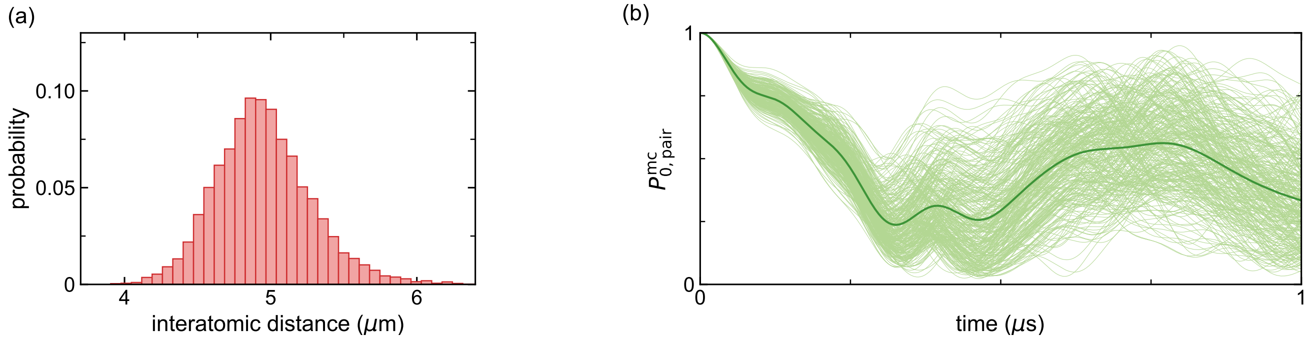

FIG. S6. **Monte Carlo samples for interatomic distance and averaged dynamics for atom pairs.** (a) The distribution of the interatomic distance after neighboring atoms with finite temperature of 4 μK are released from a 40 μK trap for 3 μs. (b) Monte Carlo simulation results for perfectly prepared (ignoring SPAM) atom pairs with the flux $\phi = 1.00(2)\pi$. Other parameters used are the same as in Fig. 2(f). The 300 independent runs and the averaged result are shown in as regular solid lines and a thick (bold) solid line, respectively.

magnification is not independently calibrated, however, we perform direct measurements of the dipolar exchange rates as a primary calibration of the dipolar interaction energies. First, we have measured the energy detuning of the triplet resonance from the single-atom resonance for the terms V_{01} and V_{30}. These measurements are performed in the actual ($\sim$5 μm spacing) tweezer configuration utilized in the main text. The results confirm the relative magnitude and signs of the expected V_{ij} exchange energies, and in combination these measurements suggest a tweezer spacing of 5.1(2) μm. However, we seek an alternative and more sensitive calibration of the dipolar exchange rate, using the technique of Ramsey coherence oscillations as employed in Ref. [6]. The procedure for this is depicted in Fig. S2. This measurement involves performing high-fidelity $\pi/2$ rotations before and after some free evolution period (t_{gap}). Because the achievement of high-fidelity $\pi/2$ pulses is difficult in the presence of strong interactions, and hence for our 5 μm tweezer spacing, we in fact perform this calibration measurement in an array that has exactly twice the spacing between neighboring atom traps (as set by the frequency tones applied to the acousto-optic deflector). We first tune the transition frequency very close to resonance, such that for singles (empty blue circles) we only observe a slow variation of the Ramsey signal (due to a slight precession of the spin during the Ramsey gap time, as the second $\pi/2$ pulse has no phase shift relative to the first pulse). For pairs, we observe an additional oscillation of the average Ramsey coherence, consistent with the coherent entangling and disentangling of the atoms at the rate V_{01}/h. This measured rate of $V_{01}^{\mathrm{cal}}/h = 0.86(2)$ MHz is 4 times smaller than the value of V as defined previously, setting $V/h = 3.44(8)$ MHz for our short-spacing arrays and likewise confirming a spacing of 4.8(1) μm based on comparison to the predictions of the Alkali Rydberg calculator (ARC) package [5].

Theoretical uncertainty estimation via Monte Carlo simulations

We perform Monte Carlo simulation to address the uncertainties of the experimentally measured/calibrated parameters, the position variation between each shot due to trap release from finite temperature, and the state preparation errors from optical pumping and STIRAP. The sampling details are summarized as following:

1. <u>The uncertainties of the Rabi rates and detunings for each transition</u>: We directly use the Ω values from the calibration fittings in Fig. S2 and the detuning uncertainty of $\sim$150 kHz from Fig. S3. For each run of the simulation, we sample all the Rabi rates and detunings from normal distributions with the measured centers and standard deviation values.

2. <u>The uncertainty of the flux phase ϕ</u>: As discussed in the text and supplement, we calibrate the flux ϕ by fitting the measured P_0 for singles at 0.525 μs [see Fig. 3(d)] with a Gaussian function. We use the fitting error of the peak position, 0.02π, as the uncertainty of the flux phase. For each run, we also sample the flux phase from a normal distribution centered at the chosen ϕ value, assuming a standard deviation of 0.02π.

3. <u>The shot-by-shot atomic position variations due to release of finite-temperature (4 μK) atoms from a 40 μK trap</u>: We follow the semi-classical method to calculate the energy distribution for optical tweezers in Refs. [1, 3], and

record the spatial positions of each atom after 3 μs release time where we generally turn on the MW pulse. To note, we assume a trap waist of 1.2 μm (based on independent calibrations), relating to radial and longitudinal trapping frequencies (for the 40 μK-deep trap) of $\omega_r/2\pi \sim 25$ kHz and $\omega_l/2\pi \sim 4$ kHz, respectively. We neglect the common-mode momentum imparted by STIRAP. The mean initial distance between two adjacent traps is taken as 4.8(1) μm from the interaction strength calibration. About the mean positions, there are thermal variations of the atomic positions in the trap. After release, the atoms move and the calculated inter-atomic distance shows a slightly broader distribution, with such a distribution for a release time of 3 μs shown in Fig. S6a. Importantly, in the relevant regime of low temperatures and low trapping frequencies prior to release, the actual distribution of calculated inter-atomic distances does not evolve much over the timescale of several μs. A release time of 3 μs is chosen as a representative time for the experimental realizations, but the distributions and their widths are largely determined by the initial (in-trap) variations and do not evolve much over 1-5 μs. For each run of the Monte Carlo simulations, we sample the atomic positions from such a distribution for each trap to evaluate the dipolar interaction strengths.

For the non-interacting single atom case, we independently run the simulation 300 times and average the obtained population dynamics for each Rydberg state. The mean values $P^{\rm mc}_{\rm n,single}$ (for state $|n\rangle$) and standard deviations from the average correspond to the solid lines and shaded regions in Figs. 2(a-c) respectively. For pairs, we account for the initial Rydberg state preparation errors. The preparation efficiency is estimated as $p = 0.98 \times 0.94 = 0.92$ from the optical pumping and STIRAP efficiencies. We first independently run the simulation 300 times for perfectly prepared atom pairs $|0\rangle \otimes |0\rangle$, and get the averaged population $P^{\rm mc}_{0,\rm pair}$ for state $|0\rangle$ and the correlation $\mathcal{C}^{\rm mc}_{00}$; see Fig. S6b. Then we calculate the population dynamics with state preparation error as $P_{0,\rm pair} = p^2 P^{\rm mc}_{0,\rm pair} + 2p(1-p)P^{\rm mc}_{0,\rm single} + (1-p)^2$. Since the single-atom dynamics should not on average contribute to the correlation function, we simply scale the correlation as $\mathcal{C}_{00} \approx p^2 \mathcal{C}^{\rm mc}_{00}$. For the 6-atom arrays, we perform 50 runs for each possible q-atom loaded pattern ($q = 1-6$), and get the averaged P_0 dynamics. Then we perform a weighted average over all possible loading cases. For the q-atom loaded cases, the weighting factor is $p^q(1-p)^{(6-q)}$. Upon performing a weighted average, we arrive at the theory curves shown in Fig. 4 of the main text.

Pair hopping rate in the strong interaction limit and its expected influence in Rydberg synthetic lattices

We consider the limit of strong interactions, i.e., $|V_{ij}| \gg |W_{ij}|$, with respect to the single-particle microwave-driven transition term $W_{ij} = \Omega_{ij}/2 = (\Omega/2)e^{i\phi_{ij}}$ and the dipole-dipole exchange term V_{ij} between the Rydberg states $|i\rangle$ and $|j\rangle$. For a pair of atoms, the microwave-driven kinetic term results in direct connections between the atom pair state $|i\rangle \otimes |i\rangle$ and the symmetric triplet state $|+\rangle = (|i\rangle \otimes |j\rangle + |j\rangle \otimes |i\rangle)/\sqrt{2}$, and likewise between $|+\rangle$ and $|j\rangle \otimes |j\rangle$. For the atom pair, each of these transitions has an enhanced coupling term of $W_p = \sqrt{2}(\Omega/2)e^{i\phi_{ij}}$. However, the intermediate state $|+\rangle$ is raised in energy by an amount $\Delta_+ = V_{ij}$ relative to the non-interacting configurations $|i\rangle \otimes |i\rangle$ and $|j\rangle \otimes |j\rangle$. Thus, applying degenerate second-order perturbation theory, one expects an effective hopping rate of bound pairs $V_{ii\to jj} \approx -W_p^2/\Delta_p = -\Omega^2 e^{i2\phi_{ij}}/(2V_{ij})$ in the strong interaction ($|V_{ij}/W_{ij}| \gg 1$) limit. Similarly, for three atoms with uniform interactions in space (e.g., arranged in a triangular pattern), one expects when $|V_{ij}/W_{ij}| \gg 1$ that three atoms can collectively hop with an effective rate of $V_{iii\to jjj} \approx -3!(\Omega/2)(\Omega/2V_{ij})^{(3-1)}e^{i3\phi_{ij}}$, consistent with the expectation [7] of an exponential increase in the timescale for co-tunneling of strings with increasing length/atom number (in the "collective" limit of uniform spatial interactions).

In considering how strong dipole-dipole interactions influence the dynamics in a many-state "Rydberg synthetic lattice," some consideration has to be made regarding the mapping between "sites of the synthetic lattice" and "physical Rydberg levels." For particular state-to-site assignments and lattice connectivities, such as that employed in Ref. [8], the interactions along the synthetic dimension are nearly local and uniform, with slight variations due to the variation of the state principal quantum numbers. This situation approximately realizes the idealized situation considered in Refs. [9, 10], where the dipolar exchange interaction between neighboring synthetic sites are local (only between nearest neighbors) and translationally invariant. In this regime, which has been studied in several works [9–11], one expects that interactions result in a rich ground state structure of highly degenerate self-bound pairs, strings, and membranes respectively for atomic dimers, 1D arrays, and 2D arrays.

In contrast, in the case of a generic assignment of internal states to synthetic lattice sites, the exchange interactions remain mostly local between neighboring synthetic sites (assuming neighbors are connected by one microwave photon and differ by at most one principal quantum number), however they can be highly non-uniform across the synthetic dimension. This variation relates to the details of the Rydberg electron orbitals involved in the exchange interaction, and results in a breaking of translation invariance for non-structured arrangements of the state-to-site assignments.

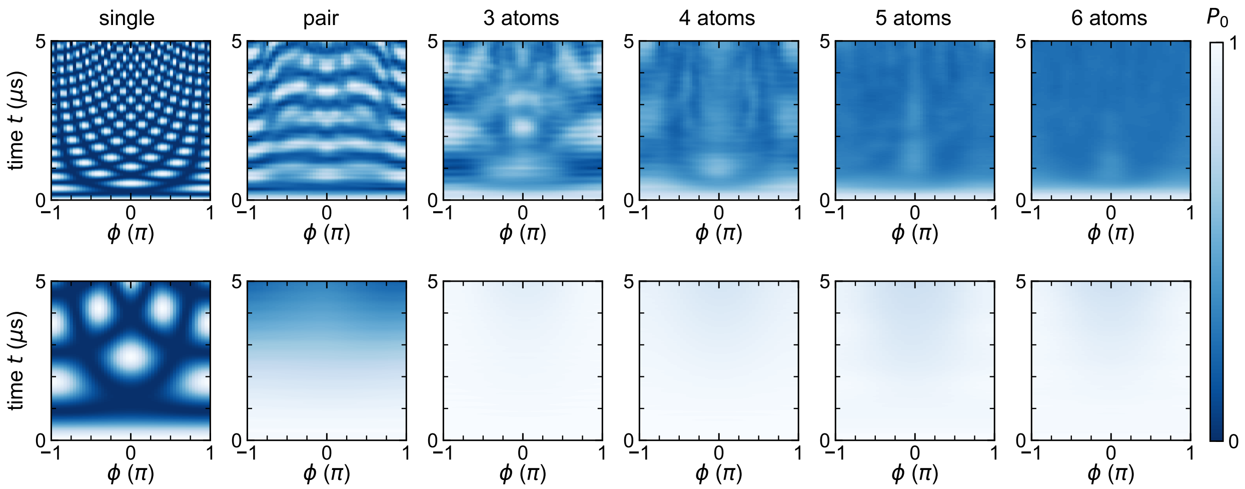

FIG. S7. P_0 **dynamics for increasing lengths of few-atom clusters (left to right), for both intermediate (top, $V/\Omega = 1.8$) and strong (bottom, $V/\Omega = 9$) interactions.** Each plot shows the phase dependence of the array-averaged P_0 dynamics. Top: $V/\Omega = 1.8$ and $\Omega/h = 1.92$ MHz, for atom array lengths from one to six. Bottom: $V/\Omega = 9$ and $\Omega/h = 0.38$ MHz, for atom array lengths from one to six. Here, only the resonant flip-flop (state conserving) dipolar interactions are considered.

For sufficiently irregular (random) arrangements of the V_{ij} terms along the synthetic dimension, one would expect (and numerics on small system sizes seem to suggest) that the atomic excitations can become localized along the synthetic dimension due to an emergent landscape of hopping disorder (or more simply due to the random interactions), similar to the scenario envisioned in Ref. [12] and somewhat reminiscent of the emergent disorder appearing in some lattice gauge theory models [13].

Finally, when considering how the renormalized pair tunneling along a single bond ($V_{ii\to jj} \approx -\Omega^2 e^{i2\phi_{ij}}/(2V_{ij})$) would modify the dynamics of bound pairs in a lattice with closed hopping pathways, or "loops," direct inspection shows that pairs would in principle experience twice the flux around a given plaquette (subject to remaining bound). However, clear signatures of this "flux enhancement" in the experiment are likely obscured by the presence of the aforementioned emergent hopping amplitude disorder.

Interactions in few-atom arrays – scrambled and frozen dynamics

In Fig. S7, we provide slightly more numerical evidence for the suggestive claims made in the main text that in our six-atom clusters we begin to see the emergence of ergodic dynamics and frozen dynamics in the regimes of intermediate and strong interactions. In the upper row of plots, we investigate over timescales of 0 to 5 μs the flux-dependent dynamics expected for atom arrays of varying size for the case of intermediate interactions ($V = 1.8\Omega$, $\Omega/h = 1.92$ MHz). In this regime, with several interaction energies V_{ij} being nearly on the same scale as the single-particle hopping terms, one may reasonably expect that the nonequilibrium dynamics of few-atom clusters becomes quite complex, with the absence of any revivals or oscillatory dynamics on reasonable timescales. This is what is observed in the six-atom calculations, where at reasonably short timescales of just a few μs there is essentially no flux dependence or dynamics to the P_0 measure, with a static value of $\approx 1/4$ found for all ϕ values. These numerical results suggest that nearly ergodic behavior may be expected in these interacting many-state systems.

In the lower row of plots of Fig. S7, we instead show the flux-dependent dynamics (over the same timescale of 5 μs) that is expected for atom arrays in the strong interaction regime ($V = 9.0\Omega$, $\Omega/h = 0.38$ MHz). In this regime, one sees that the addition of more and more atoms to the array has a very different influence on the dynamics. For pairs, the dynamics slows considerably as compared to singles and the case of pairs with intermediate interactions. For arrays with three or more atoms, the dynamics appears to nearly cease over the timescale investigated. As discussed in the main text, this is consistent with the expectation that zero-energy strings should become immobile if they are far separated in energy from the interacting configurations that would be populated by uncorrelated atom hopping in the synthetic dimension.

* These authors contributed equally to this work.
† kaden@rice.edu
‡ jcovey@illinois.edu
§ bgadway@illinois.edu

[1] J. Ang'ong'a, C. Huang, J. P. Covey, and B. Gadway, Phys. Rev. Res. **4**, 013240 (2022).
[2] N. Lorenz, L. Festa, L.-M. Steinert, and C. Gross, SciPost Phys. **10**, 052 (2021).
[3] C. Tuchendler, A. M. Lance, A. Browaeys, Y. R. P. Sortais, and P. Grangier, Phys. Rev. A **78**, 033425 (2008).
[4] T. G. Walker and M. Saffman, in Advances in Atomic, Molecular, and Optical Physics, Vol. 61 (Elsevier, 2012) pp. 81–115.
[5] N. Sibalic, J. Pritchard, C. Adams, and K. Weatherill, Computer Physics Communications **220**, 319 (2017).
[6] B. Yan, S. A. Moses, B. Gadway, J. P. Covey, K. R. A. Hazzard, A. M. Rey, D. S. Jin, and J. Ye, Nature **501**, 521 (2013).
[7] S. Raghavan, A. Smerzi, and V. M. Kenkre, Phys. Rev. A **60**, R1787 (1999).
[8] S. K. Kanungo, J. D. Whalen, Y. Lu, M. Yuan, S. Dasgupta, F. B. Dunning, K. R. A. Hazzard, and T. C. Killian, Nature Communications **13**, 972 (2022).
[9] B. Sundar, B. Gadway, and K. R. A. Hazzard, Scientific Reports **8**, 3422 (2018).
[10] B. Sundar, M. Thibodeau, Z. Wang, B. Gadway, and K. R. A. Hazzard, Phys. Rev. A **99**, 013624 (2019).
[11] C. Feng, H. Manetsch, V. G. Rousseau, K. R. A. Hazzard, and R. Scalettar, Phys. Rev. A **105**, 063320 (2022).
[12] P. Sierant, D. Delande, and J. Zakrzewski, Phys. Rev. A **95**, 021601 (2017).
[13] A. Smith, J. Knolle, D. L. Kovrizhin, and R. Moessner, Phys. Rev. Lett. **118**, 266601 (2017).
