# Supplementary material for: Strongly interacting Rydberg atoms in synthetic dimensions with a magnetic flux
Source: Nat Commun. 2024 Mar 27;15:2675. doi: 10.1038/s41467-024-46823-6 (PMC10973509; doi:10.1038/s41467-024-46823-6)
Supplement: Supplementary file 2 — Peer Review File [file 41467_2024_46823_MOESM2_ESM.pdf]

nature portfolio

Peer Review File

Strongly interacting Rydberg atoms in synthetic dimensions with a magnetic flux

Open Access This file is licensed under a Creative Commons Attribution 4.0 International License, which permits use, sharing, adaptation, distribution and reproduction in any medium or format, as long as you give appropriate credit to the original author(s) and the source, provide a link to the Creative Commons license, and indicate if changes were made. In the cases where the authors are anonymous, such as is the case for the reports of anonymous peer reviewers, author attribution should be to 'Anonymous Referee' followed by a clear attribution to the source work. The images or other third party material in this file are included in the article's Creative Commons license, unless indicated otherwise in a credit line to the material. If material is not included in the article's Creative Commons license and your intended use is not permitted by statutory regulation or exceeds the permitted use, you will need to obtain permission directly from the copyright holder. To view a copy of this license, visit http://creativecommons.org/licenses/by/4.0/.

Editorial Note: This manuscript has been previously reviewed at another journal that is not operating a transparent peer review scheme. This document only contains reviewer comments and rebuttal letters for versions considered at *Nature Communications*.

Reviewer #2 (Remarks to the Author):

The authors have thoroughly addressed my concerns. I am happy with the current paper quality with a clearer physics description as well as a sounder data analysis and presentation.

I congratulate the authors for their work and recommend it to publish on nature communication.

Reviewer #3 (Remarks to the Author):

I was already quite enthusiast with the previous version of the manuscript, and my main concern was the lack of connection between the synthetic dimension / magnetic flux and the interaction between particles. The authors have revised the manuscript to provide an additionnal paragraph satisfyingly covering this point. They also made a clear and instructive point in their detailed response to the 3 reviewers. I appreciated this very much.

I also found that the answers to the other 2 referees were sound and detailed.

I strongly recommend the acceptation of this manuscript, as it is, for publication in Nature Communication. I re-iterate my congratulations to the authors for their work.
